# Razorbills *Alca torda* in Italian Seas: A Massive Irruption of Historical Relevance and Role of Social Network Monitoring

**DOI:** 10.3390/ani13040656

**Published:** 2023-02-14

**Authors:** Rosario Balestrieri, Roberto Vento, Andrea Viviano, Emiliano Mori, Claudia Gili, Flavio Monti

**Affiliations:** 1 Dipartimento Conservazione Animali Marini e Public Engagement, Stazione Zoologica “Anton Dohrn”, Villa Comunale, 80121 Napoli, Italy; 2 Dipartimento di Scienze della Terra e del Mare, Università di Palermo, Via Archirafi 22, 90123 Palermo, Italy; 3 Consiglio Nazionale Delle Ricerche, Istituto Di Ricerca Sugli Ecosistemi Terrestri, Via Madonna del Piano 10, 50019 Sesto Fiorentino, Italy; 4 Consiglio Nazionale Delle Ricerche, Istituto Di Ricerca Sugli Ecosistemi Terrestri, Via Traversa La Crucca 3, 07100 Li Punti, Italy; 5 National Biodiversity Future Center, 90133 Palermo, Italy; 6 Department of Physical Sciences, Earth and Environment, University of Siena, Via Mattioli 4, 53100 Siena, Italy; 7 Maremma Natural History Museum, Strada Corsini 5, 58100 Grosseto, Italy

**Keywords:** auks, citizen science, irruption, Italy, Mediterranean seas, razorbill

## Abstract

**Simple Summary:**

Razorbills *Alca torda* are usually found in cold, northern waters, but hundreds of them have been detected underfed in Italy in November–December 2022. Occasional irruptions of these boreal seabirds have been documented in Italy, but poor information is available on this phenomenon. Social media (including social networks and citizen-science platforms) has allowed us to track the razorbill irruption of 2022 in detail and, in this work, we assessed the distribution of records throughout the central and the southern Mediterranean country coastlines. We collected over 250 records of this species, all of them in the western and southern Italian coasts, with some records from Malta and Maghreb and with the first record for this species in western Greece. Our work emphasizes the importance of social media, which allowed us to collect about 40% of records, to depict reliable distribution maps of rare and accidental species, attracting wildlife enthusiasts and naturalist-photographers.

**Abstract:**

Reporting on uncommon wide animal movements could help in depicting potential carry-over effects at the population level, particularly in an era of rapid climate and environmental changes. The razorbill (*Alca torda*, Linnaeus 1758) is a regular passage migrant and winter visitor to Italian seas, but with sporadic presences usually involving small numbers of individuals. Irruptions have been occasionally documented, with the last records of an unusually large number dating back to 1982. However, in the past, irruptions have only been locally reported and poorly described. Here we report on an unprecedented massive irruption of hundreds of razorbills which occurred in the central Mediterranean Sea in November-December 2022. Using citizen science platforms and photos/videos shared on social networking sites (SNSs), we estimated the relative magnitude of the irruption and described the spatial distribution of birds at sea, as well as report cases of stranded individuals. We collected a total of 267 records, both from Italy and from neighboring countries. We also discuss the likely factors affecting razorbill irruption and stress the importance of open social platforms and data sharing to aid in the early detection and estimation of such events at a wide-scale, as well as for the monitoring of the mortality of the irrupted species.

## 1. Introduction

Climatic events and the fluctuations of environmental variables may play key roles in regulating ecosystems, ultimately affecting animal movements and the spatial distribution of organisms [1]. Understanding the role of the factors affecting animal movements could help in depicting potential carry-over effects at the population level and predicting their consequences at larger scale (i.e., on ecosystems, communities and species) which have become critical to science and society, particularly in an era of rapid climate and environmental changes [2]. Consistency in animal movements is often promoted by predictable environmental factors and resource accessibility and availability [3]. Resident species can count on constant resources during the year or adapt their local movements and diet to predictable seasonal changes in food supply [4,5]. Similarly, migratory species regularly return year after year to the same breeding area and/or stopover sites and wintering grounds, taking advantage of suitable constant conditions at fixed localities along their migratory routes [6,7]. Environmental changes or habitat loss can prime individuals to switch diets or promote peculiar movement behaviours to adapt to the new conditions [8,9]. In unpredictable environments, conditions can vary greatly, eventually affecting the consistency of movement patterns and favouring the emergence of unexpected or aberrant behaviours. In such environments, e.g., desert or marine ecosystems, species have to cope with often changing feeding conditions (e.g., distribution and abundance of trophic resources) as well as with unexpected meteorological features (e.g., strong wind storms, irregular rainfall, air/water currents), resulting in largely opportunistic movements in direct response to prevailing conditions [3]. Weather-driven changes in the food supply can strongly limit animal populations and affect their fitness and survival [10].

These particular conditions may often result in irruptive phenomena, i.e., the simultaneous displacement of large numbers of individuals from their normal range [3]. These movements are usually irregular in timing and extent and are common in many species, such as boreal forest birds that feed on conifer seeds [11], rodent-eater raptors [12,13] and seabirds [14]. For instance, a massive irruption of Southern Ocean seabirds took place in the winter of 1984 off the South African coast [14]. Following the event, likely associated with a macro-scale perturbation at sea, the mortality of hundreds of individuals of several seabird species was recorded [14].

A similar event has been recorded for the razorbill *Alca torda* during the winter of 2012, when many individuals reached Florida coasts on an unprecedented scale, probably because of unseasonably warm sea surface temperatures off eastern North American coasts [15]. The razorbill is a migratory medium-size auk, widely distributed across sub-arctic and boreal waters of the North Atlantic, from eastern North America to Western Europe [16]. In Europe, it breeds primarily in Iceland, and it has increased significantly in the United Kingdom and Fennoscandia in recent years [16]. In winter, it moves away from the coast, dispersing at open waters in the North Atlantic. In recent years, spatial range shifts have been documented according to changing environmental conditions [17,18]. Poor overwinter survival prompted a displacement to more distant southerly waters of the North Sea, with razorbills foraging at a higher dietary trophic level [18]. Displacements occurring at more southern latitudes are also known, with individuals redistributing as far south as the Canary Islands and entering the western Mediterranean [19], with individuals often observed during winter months along the Spanish and French coasts [20,21]. More specifically, the phenology and population sizes of razorbills entering the Mediterranean Sea through the Strait of Gibraltar were recently described and updated [21].

In Italy (central Mediterranean Sea), the species is a regular passage migrant and winter visitor [19,22]. However, only a few observations a year are normally recorded, in the majority of cases from the northern-western sector (e.g., the Ligurian Sea) and typically involving single birds or small numbers of individuals [22,23,24]. Although, scattered (but regular), historical data are present also for more southern regions such as Lazio and Campania, among others [22].

Irruptive phenomena have rarely been documented in the past and reported only locally so far. To the best of our knowledge, large numbers have been detected only off the coasts of Puglia region for winter 1886, as mentioned by Liuzzi et al. [24], and for the Tuscan coasts in 1885, 1912, 1923, 1953 and 1981–1982 [22,25,26,27]. Nation-wide razorbill irruptions have never been described. Compared to the past, novel sources of biodiversity occurrence records are, nowadays, available, such as those originating from social networking sites (SNSs) and open platforms where records associated with images and relative metadata are shared with other users [28]. Such large amounts of information can help scientists monitor animal populations and their movements, as well as describe ecological phenomena occurring on a wide-scale [13], sometimes in near real-time. Mining citizen science data, here we report on a massive irruption of razorbills in the Italian seas that occurred in November and December 2022. An anomalous event for the country, about 40 years after the last historical record of anomalous large numbers [25,29] and of high relevance both in terms of numbers and magnitude of the irruption, with hundreds of individuals simultaneously observed in several coastal regions of the peninsula, including islands. In this work, we aimed at describing, step by step, the razorbill irruption event which occurred in the central Mediterranean Sea. We also discussed potential causes generating this phenomenon as well as relapses for biological conservation issues.

## 2. Materials and Methods

### 2.1. Data Mining on Social Networks and Citizen-Science Websites

Between November and December 2022, we conducted a data-mining campaign on social networks (Facebook, Twitter, TikTok and Instagram) to collect as much data as possible on the occurrence of razorbills in the central Mediterranean region including Italy, Corsica (FR), Malta, North Africa and Greece. Particularly, the research for occurrences was conducted on all groups dealing with birds (i.e., “EBN Italia il birdwatching italiano”, “Conoscere gli Uccelli”, “Centro Ornitologico Toscano, Soci e Simpatizzanti”) and wildlife watching (i.e., “Birdwatching & Wildlife”, “Fauna Siciliana” and “Citizen Science MUSE”). Occurrences dating November–December 2022 were confirmed by dated photos and videos of razorbills. All photographs and videos were examined and validated by expert ornithologists. Collected data were inserted in a specific dataset. Repeated observations on the same day for the same site were not retained. The authors of the records were contacted to ask to specify the exact dates, coordinates and, if possible, the number of observed individuals. We integrated our dataset through a web search for records of *Alca torda* in 2022 in the central, southern and eastern Mediterranean seas. Particularly, we searched: (i) newspaper articles on the razorbill; (ii) blogs and online forums and websites (i.e., NaturaMediterraneo, Flickr, Pinterest and YouTube); (iii) citizen-science platforms (i.e., iNaturalist, Ornitho, EBird). Search terms included all possible combinations of the words: razorbill, *Alca torda*, Italy, 2022. The same words were searched in English, Arab, Italian, Greek and French. Finally, thanks to an initiative launched by the Stazione Zoologica Anton Dohrn in collaboration with the Italian Naval League, a specific questionnaire was prepared and disseminated on the web for the collection of reports of razorbills (Supplementary File S1). Through this form, any observer was asked to report data accompanied by date, coordinates and, when possible, video/photo. The data from this form were then integrated into the database.

### 2.2. Database Construction

For data collection, we considered a limited period of three weeks, starting from the date following the first confirmed observation on 16 November 2022 off the coast of Liguria, until 8 December 2022. This approach allowed us: (i) to obtain a large number of observations from the whole peninsula following the exceptional wave of records, as the word of mouth spread and the observations were reported on the portals and on social networks; (ii) to limit the overestimation risk in both numerical and spatial terms on the longer term. Indeed, unmarked birds moving from one area to another could produce double counts and unreliable estimates [30,31]. In addition, citizen science data are subject to a series of intrinsic biases [15]. For these reasons, and waiting for standardized monitoring on a national scale that can overcome these critical issues, we have described the phenomenon only from a qualitative point of view.

To examine the at-sea spatial distribution of the razorbill, we used both a 50 × 50 and 10 × 10 km vector polygon grid shape files of the European Environment Agency (EEA) reference grid covering country borders plus a buffer of 15 km of the extent of the grid into the marine area [32,33]. Observations from the database were imported into Quantum Geographic Information System (QGIS-v. 3.6.1) and projected to the Universal Transverse Mercator (UTM) coordinate system for all spatial analyses [34]. A cell grid was considered “occupied” if at least one observation falling within the cell grid itself was reported during the study period. The resulting map thus only informs on the spatial distribution of the irruption, for the reference period. To represent the community participation in the collection of records we also plotted on the same map all records (raw data—thus, including possible multiple counts), where alive or dead birds were indicated.

In any case, given that the phenomenon was underway, these data must be considered indicative and treated with extreme caution since susceptible to possible overestimations. We then filled a dataset including an unequivocal code for each record, reporting also the date, region, coordinates, age class of the bird (juvenile vs. adult) and status (alive vs. dead) as well as the number of individuals observed. In addition, we were interested in documenting the nature of the collected data: in particular, we ranked the observations in six categories: OS (if it belonged to an ornithological survey), Social (if coming from a social network or another website of video/photo sharing), NP (if coming from newspapers), CS (data from citizen science platforms), Form (from the link sent to the Italian Naval League), or ST (from stakeholders, i.e., organizations/associations operating along the coast or at sea).

## 3. Results

### 3.1. Observation Records

**Figure 1:**
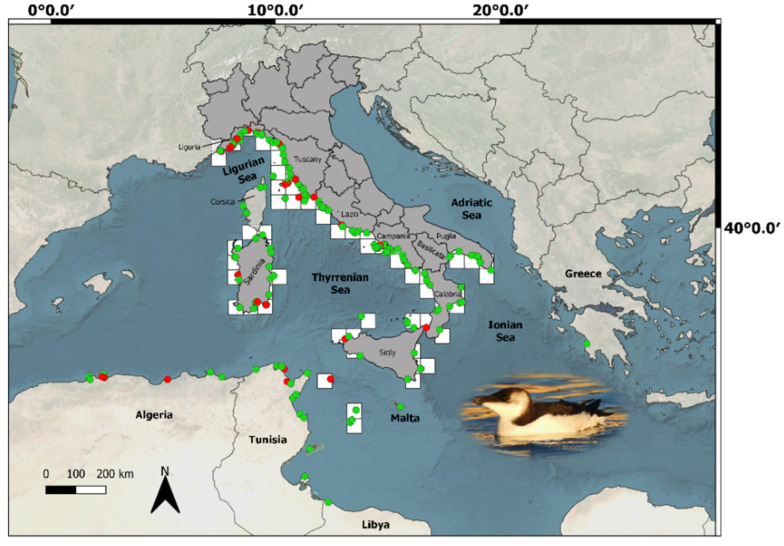
Razorbill observations during November–December 2022 in the central Mediterranean area. Dots represent the community participation in the collection of all records (including possible multiple counts), where alive (green) or dead (red) individuals are indicated. The names of countries, regions and seas are shown on the map. For Italy, the at-sea distribution is also shown for macro areas using a 50 × 50 km vector polygon grid of the EEA reference grid, where white grid cells contain at least a record (photo © Rosario Balestrieri).

**Figure 2:**
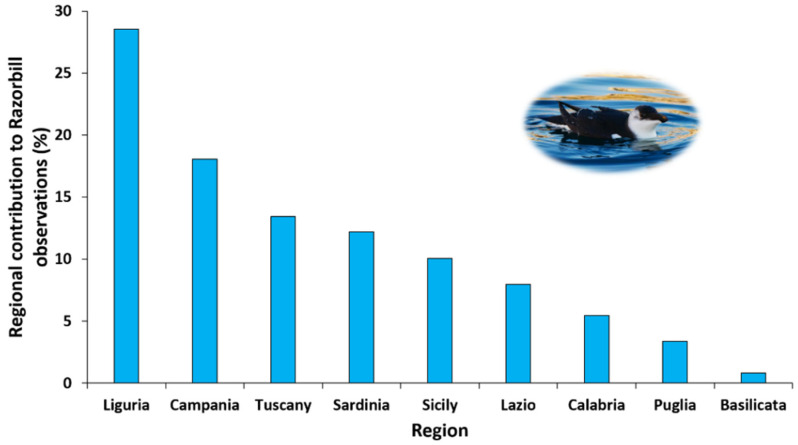
Regional contribution to razorbill observations expressed as percentage of observations over the total (*n* = 238), for Italy (photo © Rosario Balestrieri).

Between November and December 2022, 238 observations of razorbills were collected for Italy. The first confirmed sighting was of an individual recorded off the Ligurian coasts on 16 November 2022. Razorbills occurrences were documented for a total of 65 cells of the 50 × 50 km grid (Figure 1) and 127 of the 10 × 10 km grid. The at-sea spatial distribution interested nine different regions with a higher presence of the species on the west coast of the country. The majority of observations come from two main regions, Liguria and Campania (Figure 2), followed by Tuscany and Sardinia. The spatio-temporal progression of the irruption event, based on observation records, is reported in Appendix A.

The highest number of individuals recorded in a single day (on 27 November) was 747, made by 507 individuals from the Ligurian coasts plus about 212 individuals detected on the coast and wider in Tuscany, to which 28 individuals from other southern regions have been added. Two days before, on 25 November, 11 individuals were observed in the Gulf of Naples, while other observations concerned smaller flocks or single individuals across several regions. We also obtained 31 records out of Italy (Figure 1), belonging to the following countries/regions: Tunisia (*n* = 14), Algeria (*n* = 8), Corsica (*n* = 6), Malta (*n* = 1), Libya (*n* = 1) and Greece (*n* = 1). Age class for alive birds was as follows: 34.4% of records concerned only adults, 29.9% only juveniles, 11.9% mixed groups of both classes, while the remaining 23.8% concerned individuals for which the assignation by the observers of an age class was not possible (unknown).

### 3.2. Stranded Birds

During the reference period, forty razorbills were found dead (15 adults and 16 juveniles, and 9 unknown). Carcasses were retrieved along seven regions of Italy, as well as from North Africa (Figure 1). Stranded birds were brought to different museums and/or laboratories for relevant analyses. Six dead individuals were also weighed and showed low values (mean 429.8 + 98.7 g; *n* = 6). We obtained evidence of sixteen live individuals in poor health, rescued and transported to wildlife rehabilitation centers for treatment aimed at subsequent release.

### 3.3. Origin of Data

**Figure 3:**
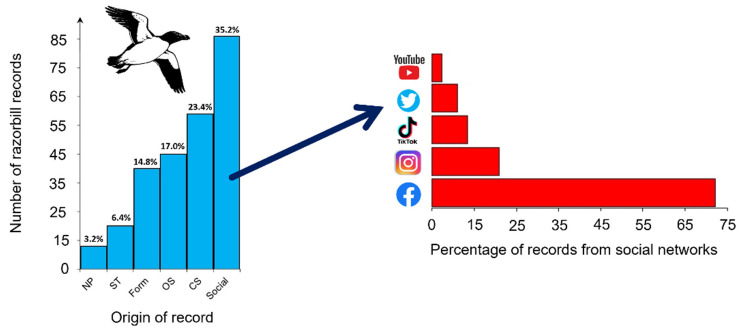
Typologies of collected records and percentage of records from different social networks and websites of video and photo sharing. Typologies of collected records were as follows: NP = newspapers; ST = stakeholders, i.e., organizations/associations operating along the coast or at sea; Form = questionnaire (Supplementary File S1); OS = ornithological survey; CS = data from citizen science platforms; Social = social network or another website of video/photo sharing.

The majority of data were reported in Social Networks (category “Social”, 35.2%) and, amongst those, on Facebook (72.4%). The category “Social” was followed by citizen science platforms (category “CS”, 23.4%) and by ornithological surveys (17.0%: Figure 3). Only two records (one European shag *Gulosus aristotelis* and one great crested grebe *Podiceps cristatus*) were incorrectly identified as razorbills.

## 4. Discussion

The razorbill irruption in winter 2022 is an event of both ecological and historical relevance. To put it into perspective, only 51 historically documented records of razorbill occurred for Liguria region between 1996 and 2005 [23], contrasting with the high number of records in 2022. More than 700 individuals were counted across the Italian coasts in a single day (27/11), a presence as massive as anomalous, never recorded before. The record of the Gulf of Naples is of relevant importance as the species had not been reported there since 1928 [35]. It is the first irruption of razorbills that seems to affect the entire peninsula, as irruptions of the past were sporadic and have been documented solely at the local scale. It should be noted that the number of ornithologists or amateurs was quite small in the past and that the use of social media as a platform to share data (video, pictures, etc.) is a relatively recent phenomenon. A limitation in recording large number of birds in the past was probably due to the lack of observers and of technological tools for data sharing. In an era of social networks and real-time data shared on web platforms, citizen science data and photos shared on Facebook mostly contributed to depicting the 2022 razorbill irruption on a national scale for the first time (Figure 1; Appendix A). However, temporal and spatial biases in opportunistically collected data should be considered in this study, including possible double counts (due to unmarked individuals moving between areas), uneven field observation effort and the use of easily accessible locations for data collection. For example, most of the observations have been reported along the coast, particularly within ports. While this could suggest that razorbills are actually selecting ports to access easier food resources (i.e., several individuals were observed targeting discarded fish from vessels), this could also be the result of uneven coverage, namely between the open sea and the coast, which may not completely reflect species occurrences and distribution. In addition, most of the observations at ports concerned young and extremely confident individuals searching for (and in some cases begging for) food as per both videos and information obtained with the form (Supplementary File S1), suggesting a shortage in food supply or suboptimal conditions. It is, thus, possible there is also a mismatch in birds’ age class and health status, between individuals attending open waters and those close to the coastline. For these reasons, a nation-scale systematic survey is urgently needed to estimate more accurately the number of individuals attending Italian waters, avoiding potential biases intrinsically associated with citizen science data. In this sense, some initiatives have already been launched in various regions of Italy with the involvement of non-governmental organizations (NGOs) and various stakeholders (e.g., the Naval League of Naples in collaboration with the Stazione Zoologica Anton Dohrn) and will likely converge at the country level.

**Figure 4:**
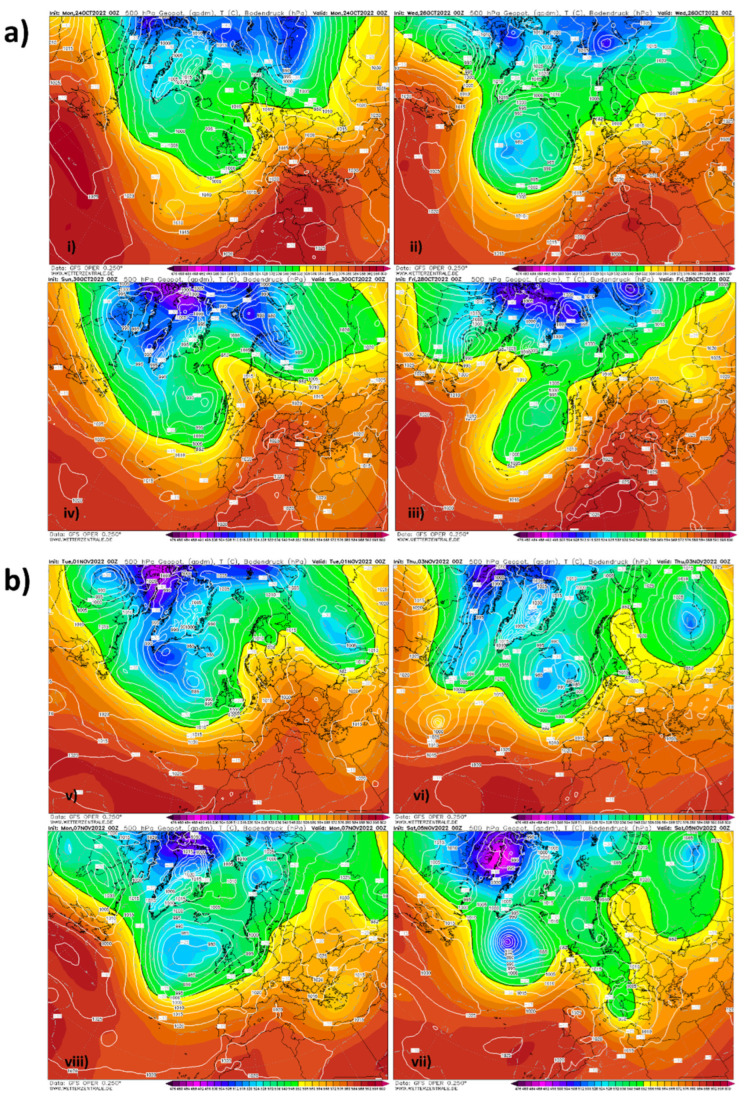
Weather conditions in the North Atlantic region corresponding to (**a**) the last decade of October 2022 (clockwise in the panel: (**i**) 24 October 2022; (**ii**) 26 October 2022; (**iii**) 28 October 2022; (**iv**) 30 October 2022) and (**b**) the first week of November 2022 (clockwise in the panel: (**v**) 1 November 2022; (**vi**) 3 November 2022; (**vii**) 5 November 2022; (**viii**) 7 October 2022) studied using the reanalysis maps of Wetterzentrale (www.wetterzentrale.de (accessed on 23 January 2023)) at 500 hPa.

The razorbill is a top predator of marine environments, tightly linked to highly productive areas of cold water upwellings where it feeds on epipelagic fishes. Lavers et al. [36] showed significant differences in razorbill annual survival rates across sites over the same time, varying with environmental conditions. It is, thus, possible that the irruption observed in 2022 could have been triggered by Atlantic storms that redistributed the contingents at sea, pushing unusual numbers of hundreds/thousands of individuals off course and considerably further south and east [37,38]. As per available weather maps and forecasts for the North Atlantic region, an exceptional concatenation of a series of deep low pressure eddies occurred off the British Isles during the end of October and first week of November, which caused strong winds that moved from the Atlantic towards the French Atlantic coasts (www.wetterzentrale.de (accessed on 23 January 2023); Figure 4). These weather events likely drove razorbills (as well as other northern seabirds) south along the Atlantic coasts of France and Portugal, until they entered the Mediterranean basin in large numbers. De la Cruz et al. [21] showed how some birds entering from the Atlantic Ocean through the Strait of Gibraltar into the Mediterranean can redistribute along the coasts of other countries, such as France. In this case, razorbills have arrived in large numbers in Italy, while their presence was recorded even at more southern and eastern latitudes such as in Malta, Tunisia, Libya and Greece. After the end of our data collection, a first record of the razorbill has also been reported on social networks for Croatia (i.e., in Veli Lošinj in January 2023). In Tunisia, Isenmann et al. [39] reported an observation of 180 individuals in the Gulf of Tunis on 23 March 1980 and an individual found dead in 1953 at Zembra, while in Greece and Libya, the current record represents the first ever sighting at the country level.

It is challenging assessing the relative causes that led to this anomalous event and how birds actually responded. Following the irruption, cases of stranded individuals have been reported from several localities. It is possible that, due to the harsh weather conditions experienced in the North Atlantic, most razorbills reached the Mediterranean already in poor conditions. It has been shown already how high-intensity North Atlantic winter cyclones can shape seabird population dynamics by affecting body condition and survival rates of the individuals (e.g., seabird mortality likely caused by starvation), sometimes leading to seabird mass-mortality events (e.g., “winter wrecks”) [38]. At the same time, one could also speculate that once in the Mediterranean, razorbills likely faced new and sub-optimal environmental conditions that could have further worsened their conditions (see mean weight in results). Lying in the warm and oligotrophic waters of the central Mediterranean, irrupted razorbills could have found very low nutrient concentrations with quite limited dynamics of significant upwellings compared to the Atlantic Ocean [40]. If compared to normal average weight of the species (ca. 650 g [41]), the low weights recorded for few dead birds seems to be in line with these hypotheses, although further in-depth investigations are needed. The observation of many individuals targeting fish discards from trawlers and fishermen’s boats within harbours seems to further support these hypotheses. Accordingly, a change in food quality associated with warmer sea temperatures compared to the North Atlantic Ocean [10] could have likely impacted these individuals. In this sense, a thorough investigation of the health status of the recovered birds (and an in-depth analysis of the dead ones) could shed light on the causes generating the irruption and suggest potential population consequences in the medium–long term.

## 5. Conclusions

Sudden changes in zoogeography and irruption events are reported to represent important clues of climatic and environmental changes [42]. Therefore, reporting on uncommon wide animal movements could help the understanding of population dynamics and complex ecological processes. Furthermore, animal irruptions may be due to lack of food [43,44], highlighting the importance of adaptive management of both wildlife and human activities (e.g., fishing).

In our case, razorbills are typical northern and boreal birds and the most recent checklist of Italian birds lists them as a regular rare overwintering species in Italy [45], mostly in the north-western regions. In addition, some irruptions by high numbers of individuals of razorbills have been documented during the last centuries, although assessing the number and distribution of birds was almost complicated by the lack of modern technologies of species recording. In the XXI century, the number of wildlife enthusiasts reporting bird observations on online platforms and social networks has exponentially increased [46,47].

In detail, new/rare species for a certain area, including alien species and accidental species, most capture the interest of photographers and enthusiasts [48]. This also increases their sharing on social networks, both for the awareness of the rarity and for the request of specific identification [49,50]. Moreover, razorbills are particularly attractive to common citizens for their resemblance to penguins, which have always stimulated empathy in humans [51]. In this context, the amount of publicly available data of razorbill occurrence in Italy in 2022 has made possible, for the first time, a detailed analysis of the irruption event, also corroborated by the confirmations of expert researchers, underlining how the Tyrrhenian and southern regions, including the islands, were all affected by the presence of some of these wintering individuals. The same data mining on social networks and citizen science online platforms has also allowed the detection of the presence of deceased individuals, probably of starvation, whose carcasses have been retrieved for specific analyses. To conclude, it would be worth stressing how most records are derived from social networks, websites of video/photo sharing and citizen-science platforms. We thus emphasize the importance of utilization of these widespread tools, followed by scientific validation of the data gathered in order to monitor species distribution and patterns of biogeographical alterations [52,53,54].

## Figures and Tables

**Figure A1:**
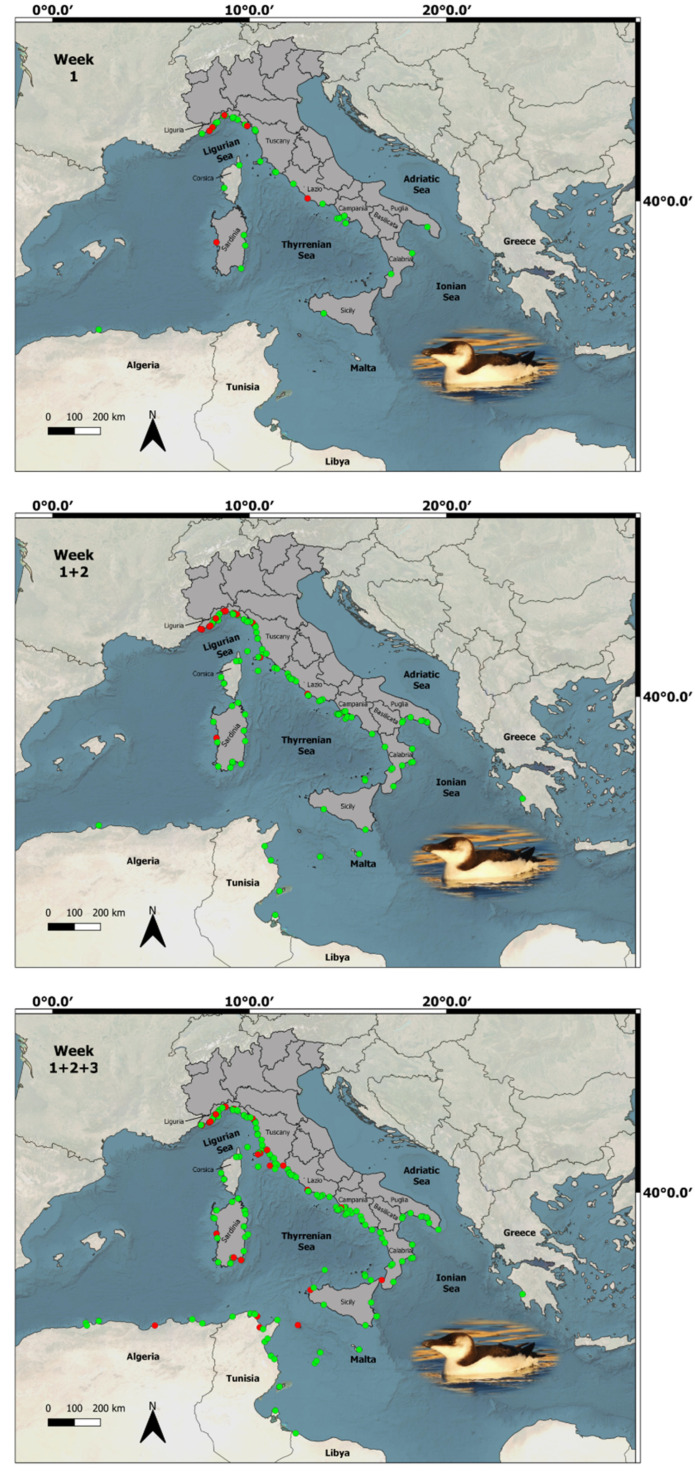
Spatio-temporal progression of the irruption event, based on observation records of Razorbill, for the 3-week period during November–December 2022 in the central Mediterranean area. The observation records are cumulatively plotted on the maps by week (Week 1; Week 1 + 2; Week 1 + 2 + 3). The dots represent the community participation in the collection of all records (including possible multiple counts), where alive (green) or dead (red) individuals are indicated. The names of countries, regions and seas are shown on the maps.

## Data Availability

All data are available via the corresponding author upon reasonable request.

## Supplementary Materials

The following supporting information can be downloaded at: https://forms.gle/kbxNnBkbdkvF4Ait6, Supplementary File S1: The form “Razorbills *Alca torda* in Italian Seas” (accessed on 8 December 2022).

## Appendix A

Spatio-temporal progression of the irruption event.
